# Ultra-high-frequency ECG volumetric and negative derivative epicardial ventricular electrical activation pattern

**DOI:** 10.1038/s41598-024-55789-w

**Published:** 2024-03-07

**Authors:** Pavel Leinveber, Josef Halamek, Karol Curila, Frits Prinzen, Jolana Lipoldova, Magdalena Matejkova, Radovan Smisek, Filip Plesinger, Andrej Nagy, Miroslav Novak, Ivo Viscor, Vlastimil Vondra, Pavel Jurak

**Affiliations:** 1 International Clinical Research Center, St. Anne’s University Hospital Brno, Brno, Czech Republic; 2 Institute of Scientific Instruments, The Czech Academy of Sciences, Brno, Czech Republic; 3 Cardiocenter, Third Faculty of Medicine, Charles University and University Hospital Kralovske Vinohrady, Prague, Czech Republic; 4 Department of Physiology, Cardiovascular Research Institute Maastricht, Maastricht University, Maastricht, Netherlands; 5 First Department of Internal Medicine and Cardioangiology, St. Anne’s University Hospital Brno, Brno, Czech Republic; 6 Faculty of Medicine, Masaryk University, Brno, Czech Republic; 7 Department of Biomedical Engineering, Faculty of Electrical Engineering and Communication, Brno University of Technology, Brno, Czech Republic

**Keywords:** Cardiac device therapy, Biomedical engineering

## Abstract

From precordial ECG leads, the conventional determination of the negative derivative of the QRS complex (ND-ECG) assesses epicardial activation. Recently we showed that ultra-high-frequency electrocardiography (UHF-ECG) determines the activation of a larger volume of the ventricular wall. We aimed to combine these two methods to investigate the potential of volumetric and epicardial ventricular activation assessment and thereby determine the transmural activation sequence. We retrospectively analyzed 390 ECG records divided into three groups-healthy subjects with normal ECG, left bundle branch block (LBBB), and right bundle branch block (RBBB) patients. Then we created UHF-ECG and ND-ECG-derived depolarization maps and computed interventricular electrical dyssynchrony. Characteristic spatio-temporal differences were found between the volumetric UHF-ECG activation patterns and epicardial ND-ECG in the Normal, LBBB, and RBBB groups, despite the overall high correlations between both methods. Interventricular electrical dyssynchrony values assessed by the ND-ECG were consistently larger than values computed by the UHF-ECG method. Noninvasively obtained UHF-ECG and ND-ECG analyses describe different ventricular dyssynchrony and the general course of ventricular depolarization. Combining both methods based on standard 12-lead ECG electrode positions allows for a more detailed analysis of volumetric and epicardial ventricular electrical activation, including the assessment of the depolarization wave direction propagation in ventricles.

## Introduction

Information about the ventricular electrical activation pattern is important in the diagnostics of ventricular conduction abnormalities. Standard 12-lead ECG markers such as QRS duration and morphology are insufficient for its precise assessment. Nevertheless, obtaining the information from the standard 12-lead ECG represents the simplest and most convenient way regarding daily clinical practice.

The Ultra-High-Frequency ECG (UHF-ECG) methodology describes the pattern of electrical depolarization of ventricles by standard 12-lead ECG electrode positions on the thorax but using the information from a large range of frequencies in the QRS complex^1,2^. These QRS frequency oscillations are temporally and spatially sensitive and can be registered by the precordial leads above the activated myocardium. Recently, the ex-vivo experimental study showed that the UHF-ECG information about ventricular electrical activation has a volumetric character^3^. UHF-ECG has been shown to have diagnostic potential in cardiac pacing areas like cardiac resynchronization therapy (CRT)^4,5^, His bundle, Left bundle branch, and right ventricular pacing^6,7^.

The electrical epicardial depolarization of ventricles can be assessed using the concept called “intrinsic deflection”, first introduced by Lewis et al. in 1915^8^. The term refers to the steep downward deflection of the electrogram, recorded by the unipolar exploring electrode, indicating the epicardial breakthrough of the electrical depolarization wave located directly under the exploring electrode^9^. The maximum downslope is considered the most accurate marker of local tissue activation^10^ and a cornerstone of electrocardiographic imaging (ECGi)^11^. The analogous relationship between the direct epicardial depolarization and the signal from precordial ECG leads is called “intrinsicoid (semi-intrinsic) deflection”^12^. The evaluation of the epicardial electrical ventricular activation, using the intrinsicoid deflection design, has been recently reported^13,14^ to improve the selection criteria for CRT.

During normal physiological activation, ventricular walls are activated from the endocardium to the epicardium. However, during ectopic activation, this may be different, such as during bundle branch blocks and epicardial ventricular pacing. A better understanding of this transmural course of activation may be helpful to better diagnose the conduction abnormality in a patient.

The present study was designed to compare the volumetric and epicardial activation derived from the UHF-ECG and ND-ECG methods, respectively, and to test the hypothesis that the combination of the volumetric and epicardial activation assessment allows more detailed insight into the electrical depolarization wave in ventricles using the 12-lead ECG.

## Methods

### Subjects

We retrospectively analyzed 390 records derived from data collected prospectively in a research project aimed at assessing electrical ventricular depolarization using the Ultra-High-Frequency ECG (UHF-ECG) methodology. This investigation took place at St. Anne’s University Hospital Brno, Czech Republic. Data included 5 min measurements of 12-lead ECG signals with a sampling frequency of 5 kHz and amplitude resolution of 3 nV. The records were divided into three groups of subjects: healthy subjects with normal ECG (N = 169), LBBB patients (N = 153), and right bundle branch block (RBBB) patients (N = 68). The normal ECG group includes healthy volunteers with normal ECG morphology and QRS duration ≤ 115 ms. The LBBB subjects were recruited from a cohort of heart failure patients with left ventricular ejection fraction (LVEF) lower than 35%, clinically identified for cardiac resynchronization therapy (CRT). Inclusion criteria was “strict” LBBB morphology defined by Strauss criteria (QRS duration ≥ 130 ms in women and ≥ 140 ms in men, QS or rS in leads V1 and V2, and mid-QRS notching or slurring in ≥ 2 of leads I, aVL, V1, V2, V5 or V6)^15^. RBBB group included 51 heart failure patients indicated for CRT and/or implantable cardioverter-defibrillator (ICD) and 17 patients with chronic RBBB, ischemic heart disease, and cardiomyopathy. All RBBB subjects fulfilled the criteria for complete RBBB defined as QRS duration ≥ 120 ms, broad, notched secondary R waves (rsr′, rsR′, or rSR′ patterns) in right precordial leads (V1 and V2), and wide, deep S waves of greater duration than R wave or greater than 40 ms in leads I and V6^16^. Above these groups, three CRT patients were selected to demonstrate the effect of ventricular pacing on depolarization maps. The study was approved by the Ethics Committee of St. Anne’s University Hospital Brno, all methods were performed in accordance with relevant guidelines and regulations, and all patients gave their written informed consent.

### UHF-ECG volumetric depolarization pattern analysis

**Figure 1:**
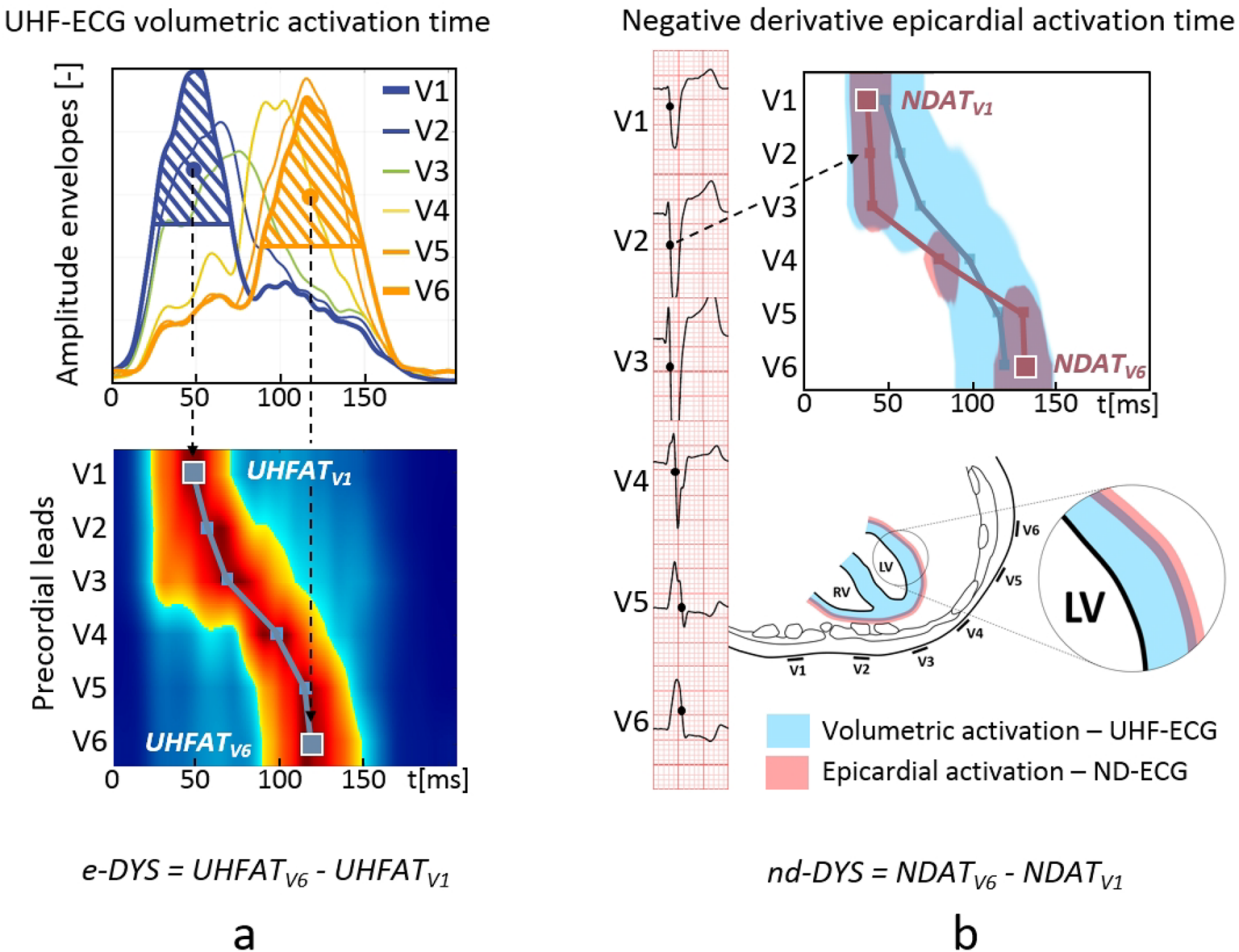
Methodological concept of assessment of ventricular electrical dyssynchrony in (**a**) UHF-ECG and (**b**) ND-ECG in LBBB subject with e-DYS 71 ms and nd-DYS 94 ms. The local activation time by the UHF-ECG (UHFAT) in each lead is defined as the center of gravity of the amplitude envelope, neglecting the low-level signal (below 0.5 of the amplitude maximum). The local activation time by the ND-ECG (NDAT) corresponds to the maximum of a normalized negative derivative component of the QRS complex that refers to its maximal downslope. The dark blue line is created by connecting the UHFAT and the dark red line by NDAT points. The zero timeline indicates the beginning of the QRS complex. *LBBB* left bundle branch block, *e-DYS* electrical interventricular dyssynchrony by the UHF-ECG method, *nd-DYS* electrical interventricular dyssynchrony by the ND-ECG method, *UHFAT* UHF activation time, *NDAT* ND activation time.

The UHF-ECG analysis has been described elsewhere^1^. Briefly, QRS complexes were detected, and amplitude passband envelopes in sixteen frequency bands (150–250; 200–300;...; 900-1000Hz) were computed using Hilbert transformation. QRS envelopes were then averaged according to the R-wave trigger, normalized in 16 frequency bands, and averaged over all frequency bands. Averaged QRS envelopes in precordial leads (V leads) define the time-spatial distribution of electrical myocardial activation and are used to plot ventricular depolarization maps (Fig. 1a.). Local UHF-ECG activation time (UHFAT) in each lead is defined as the center of gravity of the averaged QRS envelope, neglecting the low-level signal (below 0.5 of the amplitude maximum). UHF-ECG ventricular depolarization maps are created by arranging the precordial leads from V1 to V6 and color-coding the level of activity as originally described in^1,2^. The dark blue line in the depolarization map (Fig. 1a) represents the activation-specific depolarization pattern created by connecting UHFAT among all V leads.

### ND-ECG epicardial depolarization pattern analysis

Defining the exact point on the intrinsicoid deflection that corresponds to the epicardial activation time is not unequivocal^17,18^. Similarly to^19^ or^20^, and for its definition clarity, we applied the negative derivative approach. The local ventricular activation time was computed as the point of maximum negative derivative (−dV/dtmax) that corresponds to the steepest downslope of the QRS segment of a precordial ECG lead (Fig. 1b). This was determined in the standard ECG frequency band (0.05–100 Hz) of the averaged QRS complex. The positive derivative component was zeroed, and normalized signal −dV/dt was used to plot the ND epicardial activation pattern (Fig. 1b, dark red line). The local activation time (NDAT) was defined as the maximum of a normalized derivative signal. The dark red line in Fig. 1b represents the activation-specific depolarization pattern created by connecting NDAT among all V leads.

For easier comparison of both UHF-ECG and ND-ECG depolarization patterns, we have created a simplified volume–epicardial relation in one map (Fig. 1b). The light blue color represents a UHF-ECG depolarization above 50% of an amplitude envelope, while the dark red color depicts the ND-ECG steepness of the downslope with the threshold of 50% in each V lead.

### Dyssynchrony analysis

The UHF-ECG interventricular electrical dyssynchrony (e-DYS) was defined as the difference between the earliest and latest local activation (UHFAT) in V leads (Fig. 1a). Analogously, the ND-ECG interventricular electrical dyssynchrony (nd-DYS) was computed as the difference between the earliest and latest local activation (NDAT) in V leads (Fig. 1b). The positive e-DYS and the nd-DYS value corresponds to late activation of the left lateral wall like in LBBB. Global QRS duration (QRSd) was measured fully automatically using the custom-made software originated from^21^ and was inspected by a skilled cardiologist for errors. The relationships between dyssynchrony parameters e-DYS, nd-DYS, and QRSd were assessed by the Pearson correlation coefficient. The relationship between UHF-ECG and ND-ECG depolarization patterns determined by local activation times was also analyzed using the Pearson correlation coefficient. The numerical results are given as median [25th and 75th percentile].

## Results

**Figure 2:**
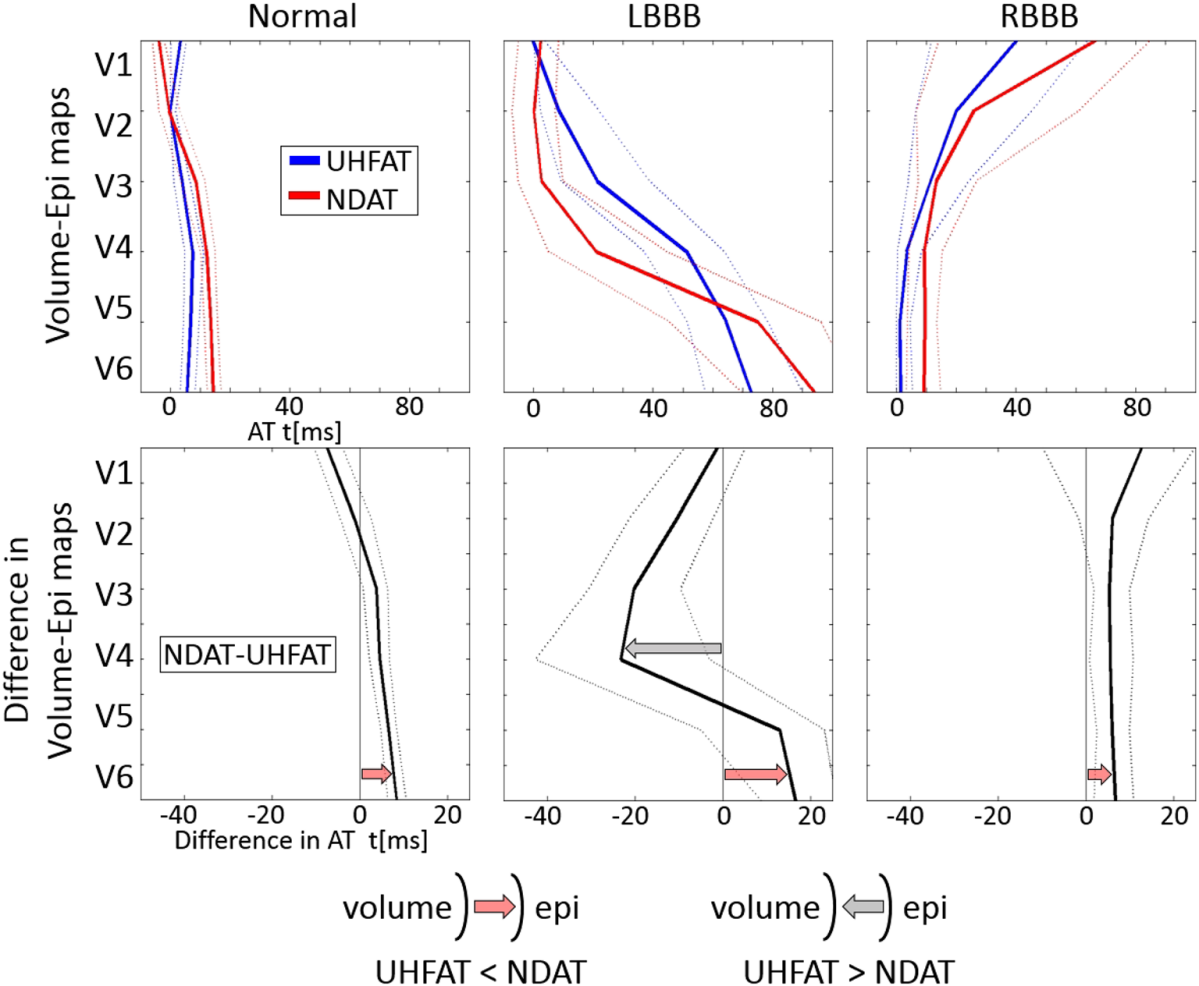
Average ventricular depolarization patterns (top) and differences between the methods (bottom) for the subject groups. UHFAT is represented by the blue color and NDAT by the red color. The thick lines represent the median and the thin lines the 25th and 75th percentile. Red and grey arrows indicate the transmural direction of the depolarization wave. The first UHFAT activated segment is placed to 0 ms in the upper row. *AT* activation time, *RBBB* right bundle branch block, other abbreviations as in Fig. 1.

The average ventricular depolarization patterns from UHF-ECG and ND-ECG (Fig. 2, top) and the differences between both methods (Fig. 2, bottom) for all three groups are depicted in Fig. 2. In each group, both average depolarization patterns show a general similar direction of the ventricular electrical activation between V1 and V6. In the Normal group, the ventricular depolarization is fast and synchronous, but the epicardial activation (NDAT) is overall delayed behind the volumetric activation (UHFAT). LBBB’s global activation pattern shows a considerable delay of the left lateral wall (V4–V6) against the right ventricle (RV) and septum (V1–V3). The earliest ventricular depolarization occurs in V2–V3 as an NDAT followed by delayed UHFAT but this epi-volume activation pattern twists in V5 and V6 (Fig. 4a). The global activation sequence in RBBB is analogically opposite to the LBBB. The average NDAT activation pattern is delayed in all precordial leads compared to UHFAT.

**Figure 3:**
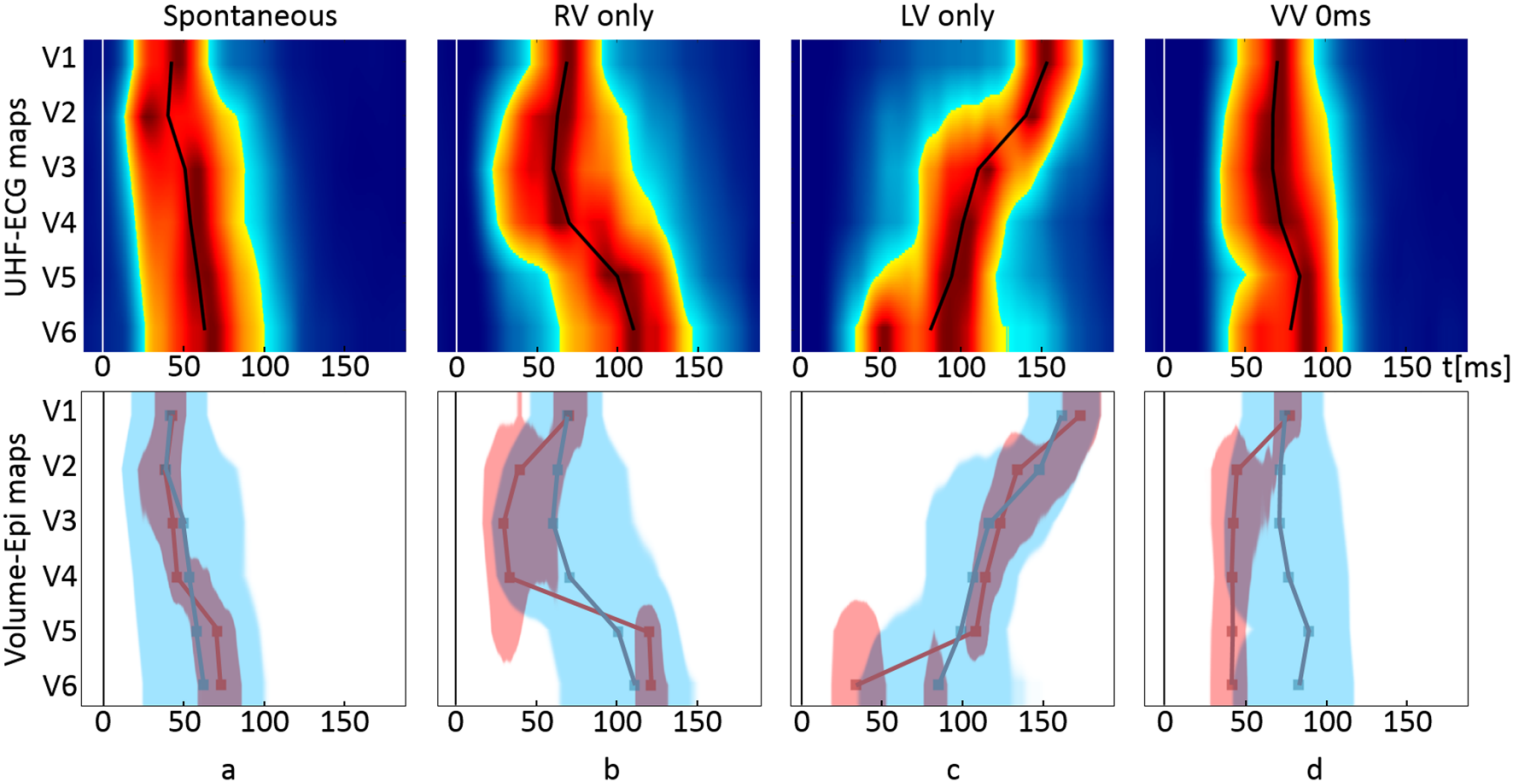
Example of the NIVCD CRT recipient suffering from DCM, QRSd 130ms, AVB 1st degree, EF 30%, NYHA III, implanted with the bi-ventricular pacemaker during various pacing scenarios. The LV electrode is placed in the mid posterolateral branch of the coronary sinus, and the RV electrode is placed in the RV apex. The top row represents UHF-ECG maps, and the bottom row shows the volume–epicardial (light blue–dark red) relation. The lines in the maps are created by connecting the UHFAT and NDAT points over the V leads. The narrow upright line at zero timeline indicates the pacing peak or the beginning of the QRS complex. (**a**) Spontaneous rhythm, (**b**) only the RV electrode stimulates (RV only), (**c**) only the LV electrode stimulates (LV only), (**d**) simultaneous stimulation by the RV and LV electrodes and ventricle-ventricle (VV) set to 0 ms. *NIVCD* non-specific interventricular conduction delay, *CRT* cardiac resynchronization therapy, *DCM* dilated cardiomyopathy, *QRSd* QRS duration, *AVB* atrioventricular block, *EF* ejection fraction, *NYHA* New York Heart Association class, *LV* left ventricle, *RV* right ventricle, *UHFAT* UHF activation time, *NDAT* ND activation time.

**Figure 4:**
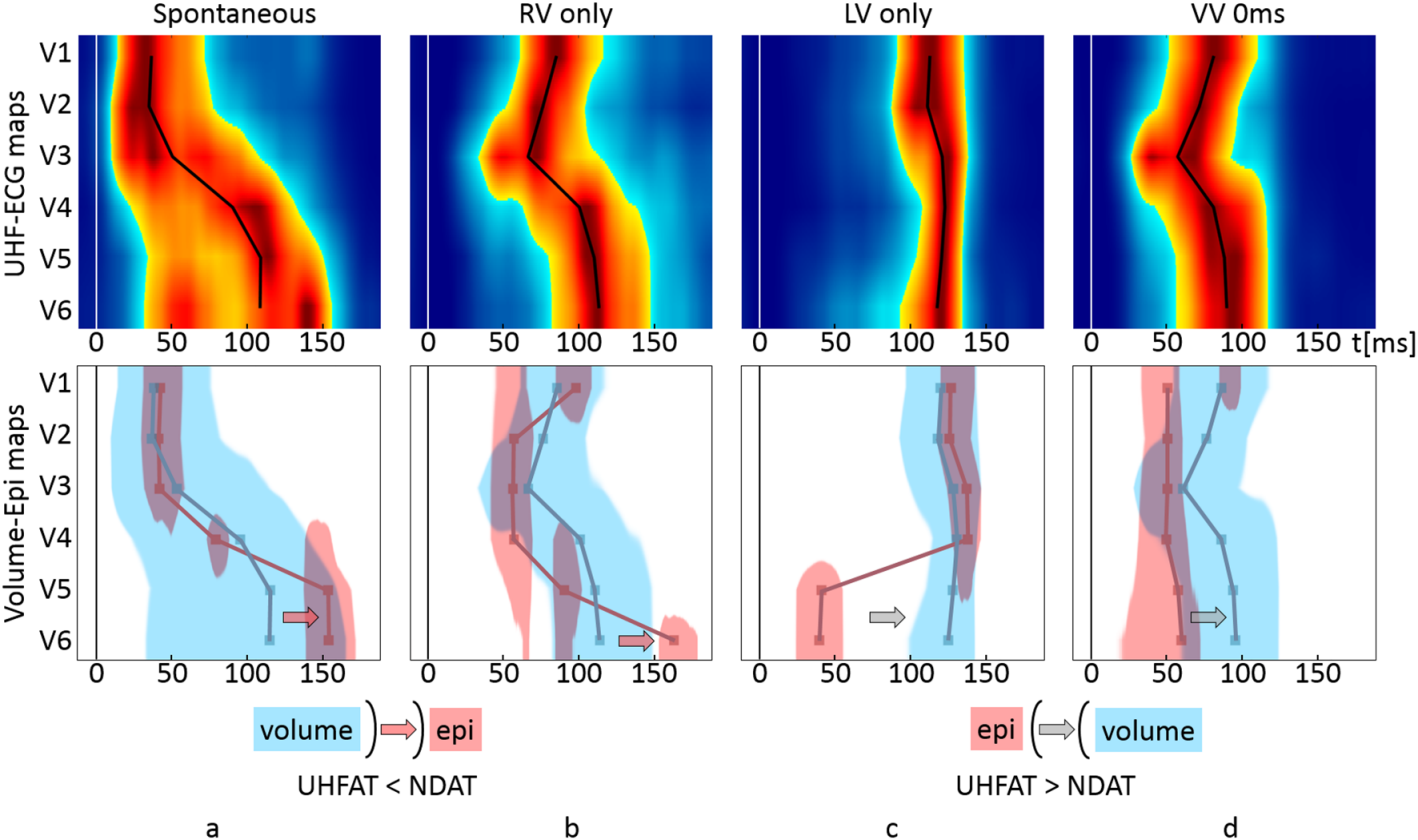
Example of the LBBB CRT recipient suffering from DCM, QRSd 169ms, EF 35%, NYHA II, implanted with the bi-ventricular pacemaker during various pacing scenarios. The LV electrode is placed in the mid posterolateral branch of the coronary sinus, and the RV electrode is placed in the RV apex. The top row represents UHF-ECG maps, and the bottom row shows their mutual volume–epicardial (light blue–dark red) relation. The lines in the maps are created by connecting the UHFAT and NDAT points over the V leads. The narrow upright line at zero timeline indicates the pacing peak or the beginning of the QRS complex. (**a**) Spontaneous rhythm, (**b**) only the RV electrode stimulates (RV only), (**c**) only the LV electrode stimulates (LV only), (**d**) simultaneous stimulation by the RV and LV electrodes and ventricle-ventricle (VV) set to 0 ms. *LBBB* left bundle branch block, other abbreviations as in Fig. 3.

**Figure 5:**
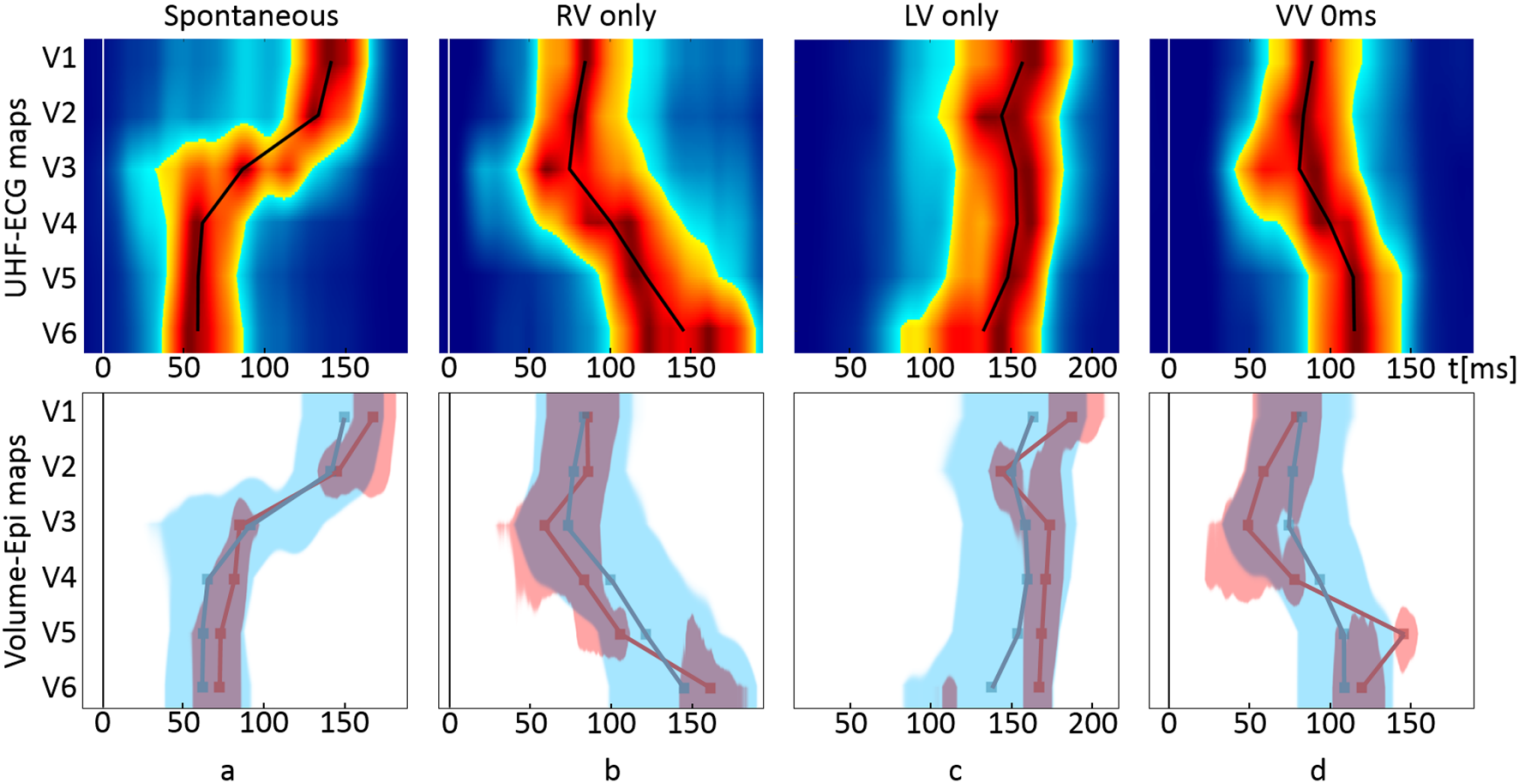
Example of the RBBB CRT recipient suffering from chronic heart failure (a consequence of valvular disease), QRSd 178 ms, persistent atrial fibrillation, EF 25%, NYHA III, implanted with the bi-ventricular pacemaker during various pacing scenarios. The LV electrode is placed in the basal posterolateral branch of the coronary sinus, and the RV electrode is placed in the RV apex. The top row represents UHF-ECG maps, and the bottom row shows their mutual volume–epicardial (light blue–dark red) relation. The lines in the maps are created by connecting the UHFAT and NDAT points over the V leads. The narrow upright line at zero timeline indicates the pacing peak or the beginning of the QRS complex. (**a**) Spontaneous rhythm, (**b**) only the RV electrode stimulates (RV only), (**c**) only the LV electrode stimulates (LV only), (**d**) simultaneous stimulation by the RV and LV electrodes and ventricle-ventricle (VV) set to 0 ms. *RBBB* right bundle branch block, other abbreviations as in Fig. 3.

To accentuate the spatio-temporal differences between UHF-ECG and ND-ECG depolarization maps, we have included the depolarization patterns from three examples of CRT recipients during various pacing scenarios—Spontaneous rhythm, left ventricle (LV)-only pacing, RV-only pacing, and biventricular pacing from both leads at the same time (Figs. 3, 4, 5).

Figure 3 presents the patient with a non-specific interventricular conduction delay (NIVCD) and implanted biventricular pacemaker. During the RV-only stimulation (Fig. 3b), the first activation emerges by the epicardial NDAT under V2–V4, followed by volumetric UHFAT, and later by the delayed LV lateral wall under V5–6. The LV-only pacing first activates under V6 in both maps but much earlier epicardially by NDAT (Fig. 3c). Simultaneous RV and LV pacing (Fig. 3d) links both preceding separate stimulations and shows the resulting bi-ventricular paced depolarization maps.

In the LBBB CRT recipient (Fig. 4), UHF-ECG and ND-ECG patterns during RV-only pacing are similar to NIVCD patterns, with the difference in very late NDAT activation under V6 in the LBBB patient (Fig. 4b). When LV-only pacing is provided (Fig. 4c), the epicardial NDAT under V5-6 is very premature against the volumetric UHFAT.

In the RBBB patient (Fig. 5), RV apical pacing reverses the pattern of the RBBB activation into an LBBB shape of depolarization. The soonest depolarization occurs under the V3 by epicardial NDAT. In this RBBB case, the LV electrode is placed in the basal posterolateral branch of the CS. During the LV-only pacing, the depolarization wave travels from the basal part of the LV towards the V6 lead, which is located laterally against the LV. This effect likely results in delayed epicardial NDAT activation in V6.

The numerical parameters e-DYS, nd-DYS, and QRSd reflecting the division into groups of subjects are in Table 1. The values of the interventricular electrical dyssynchrony parameters calculated by the ND-ECG method (nd-DYS) are higher than those calculated by the UHF-ECG method (e-DYS) in every of all observed groups. The overall difference between e-DYS and nd-DYS medians is approximately 11ms in the Normal group but 23ms in the bundle branch block (BBB) groups.

**Table 1:** Numerical parameters e-DYS, nd-DYS, and QRSd in different groups of subjects.

	Normal (N = 169)	LBBB (N = 153)	RBBB (N = 68)
e-DYS (ms)	8 [5; 11]	73 [58; 90]	-45 [-66; -23]
nd-DYS (ms)	19 [17; 21]	96 [77; 111]	-68 [-85; -20]
QRSd (ms)	93 [88; 99]	174 [162; 184]	158 [143; 170]

The values are given as median [25th and 75th percentile]. *Normal* normal ventricular electrical conduction, *LBBB* left bundle branch block, *RBBB* right bundle branch block, *e-DYS* electrical interventricular dyssynchrony by UHF-ECG method, *nd-DYS* electrical interventricular dyssynchrony by ND-ECG method, *QRSd* QRS duration.

**Figure 6:**
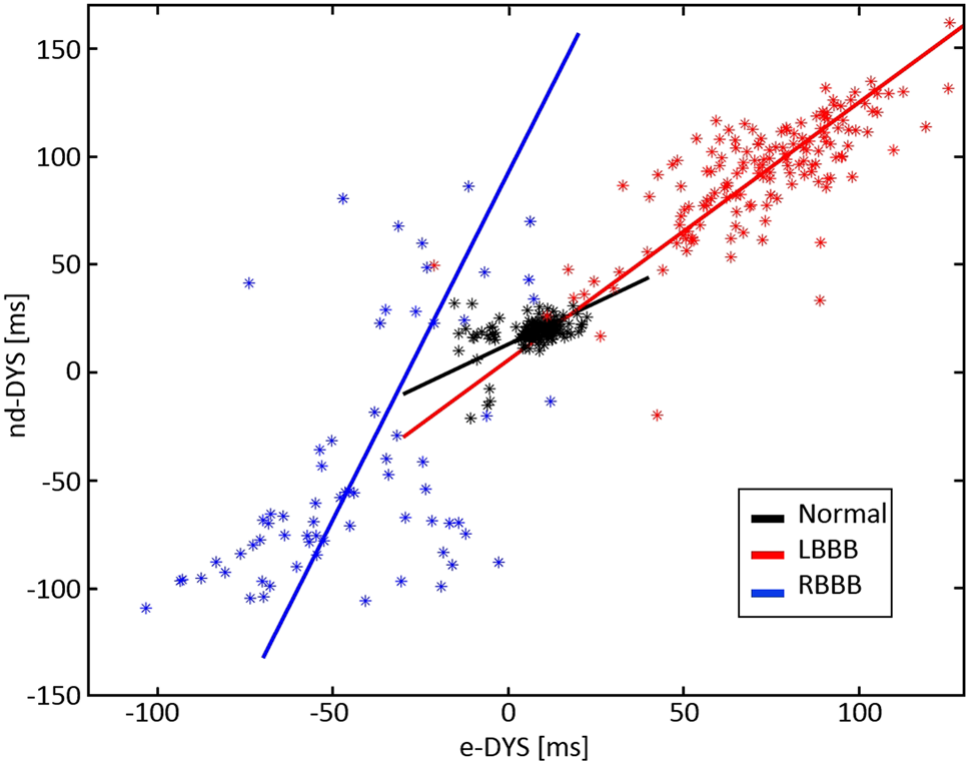
The scatter plot depicts the relation between e-DYS and nd-DYS parameters. Color coding of the groups: normal black, LBBB red, and RBBB blue. Abbreviations as in Table 1.

The scatter plot in Fig. 6 shows the relation between the e-DYS and nd-DYS numerical parameters. Correlation coefficients between e-DYS, nd-DYS, and QRSd are in Table 2, together with the parameters of orthogonal regression analysis, *nd-DYS* = K1*e-DYS + *K0*, and the correlation between UHFAT and NDAT depolarization patterns. The very high correlation over All data (e-DYS vs. nd-DYS) is given by the specific conditions of each of the three groups. The interplay of the high positive dyssynchrony values in the LBBB group, high negative dyssynchrony values in the RBBB group, and very low values from the Normal group contribute to a robust correlation (Fig. 6). The lower correlation between e-DYS and nd-DYS in the Normal group is due to the physiologically low value of the dyssynchrony reflecting the direction and speed of the wavefront propagation. Also, the lower correlation coefficients between both dyssynchrony parameters and QRS are affected by the fact that, unlike the QRS parameter, the dyssynchrony parameters can also be negative. Overall, very strong correlations between the activation time shapes (UHFAT vs. NDAT) were observed.

**Table 2:** Correlation analysis of the numerical parameters (e-DYS, nd-DYS, and QRSd) and the depolarization patterns (UHFAT vs NDAT) over all the data and ventricular conduction groups.

Correlation coefficient	All data (N = 390)	Normal (N = 169)	LBBB (N = 153)	RBBB (N = 68)
e-DYS vs nd-DYS	0.916 [0.898; 0.931]	0.382 [0.246 0.504]	0.752 [0.674; 0.814]	0.537 [0.342; 0.687]
e-DYS vs QRSd	0.429 [0.344; 0.507]	− 0.182 [− 0.324; − 0.032]	0.548 [0.426; 0.650]	− 0.475 [− 0.641;− 0.266]
nd-DYS vs QRSd	0.365 [0.276; 0.448]	0.067 [− 0.085; 0.216]	0.489 [0.358; 0.601]	− 0.502− [− 0.662; − 0.300]
UHFAT vs NDAT	0.829 [0.620; 0.914]	0.709 [0.459; 0.860]	0.879 [0.808; 0.928]	0.858 [0.433; 0.965]
K1 (n.u.)	1.25 [1.23; 1.17]	0.78 [0.59; 0.99]	1.17 [1.10; 1.26]	3.16 [2.80; 3.65]
K0 (ms)	6.21 [4.90; 7.25]	12.9 [11.0; 14.4]	7.05 [0.56; 12.4]	93.1 [78.9; 113.7]

K1 and K0 are parameters of the orthogonal regression nd-DYS = K1*e-DYS + K0. The values are given as median [25th and 75th percentile]. *UHFAT* UHF activation time, *NDAT* ND activation time, and other abbreviations as in Table. 1.

## Discussion

The simple and reliable assessment of the electrical ventricular depolarization represents an important diagnostic need in cardiac electrophysiology, especially in cardiac pacemaker therapy. Unfortunately, current clinical methods do not offer easy and reliable solutions apart from a standard reading of QRS morphology and its duration. The present study introduces simple but still advanced information about the propagation of electrical ventricular depolarization. The novelty lies in the combination of the two independent approaches using a standard 12-lead ECG electrode placement.

### Interventricular dyssynchrony parameters

nd-DYS, e-DYS, and QRSd are ECG-based parameters that can be objectively determined. nd-DYS and e-DYS parameters directly assess the ventricular dyssynchrony, while the QRSd is rather a surrogate of the dyssynchronous electrical ventricular depolarization. Both e-DYS and nd-DYS measure interventricular dyssynchrony directly in milliseconds, but each carries specific information. While e-DYS has been used previously^1,4,7^, we established the nd-DYS dyssynchrony parameter by utilizing the “intrinsicoid deflection” method similarly as in^19,20^. nd-DYS values are higher than e-DYS values, both in the entire cohort and in the three separate groups. UHF-ECG signals determine the ventricular activation from the depolarized volume under the electrode, while the ND-ECG represents epicardial depolarization. Jurak et al. recently proved the volumetric character of the UHF-ECG by putting in context the direct epicardial, transmural, and body surface electrograms on an experimental animal model^3^. The ventricular dyssynchrony measured by the UHF-ECG methodology in Jurak’s study was significantly lower than direct epicardial measurement on the heart surface. This experimental finding corresponds well with the volume (UHF-ECG) versus epicardial (ND-ECG) dyssynchrony measurement findings in the current study.

A significant correlation between e-DYS and nd-DYS enables one to choose either of both parameters to describe interventricular dyssynchrony by a numerical parameter. Nevertheless, a single parameter cannot depict the specifics of conduction irregularities. Such details can be described by the UHF-ECG depolarization maps, and before all, by their simultaneous volume-epi analysis (Figs. 3, 4, 5).

### UHF-ECG and ND-ECG ventricular depolarization patterns

UHF-ECG and volume-epi depolarization maps describe the specific time-spatial distribution of electrical activation in ventricles. Computed UHF and ND activation times in the areas under the V leads create ventricular depolarization shapes. Each method carries different information associated with the volumetric and epicardial character of the analyzed electrical conduction. The difference between UHFAT and NDAT activation times under the specific V leads can identify the propagation direction of the depolarization wave.

In physiological ventricular conduction (Normal group), the ventricular electrical depolarization is very fast and synchronous (narrow and strait depolarization map between V1 and V6). Differences between the methods are thus small but still evident. Early NDAT in V1 and V2 reflects the earliest epicardial breakthrough in the anterior RV wall^22,23^. The very early onset of the epicardial activation in the anterior RV wall is due to the thinness of the RV wall allowing the rapid transmission to the epicardium of the later endocardial wavefront in the RV^23^. Overall, the UHFAT volumetric activation precedes the NDAT epicardial activation indicating standard “endo-to-epi” depolarization through the conduction system.

In the LBBB, RV is activated normally through the right bundle branch, while the septum depolarizes inversely, progressing from right to left^15^. Because the RV activation follows the standard pathway, including the moderator band, the initial depolarization becomes apparent epicardially in the right precordial leads (V1–V3). Slow depolarization wave propagation by myocardial conduction across the interventricular septum^24^ increases the time difference between the UHFAT and NDAT in V2–V4 areas. When a critical mass of the LV myocardium is finally depolarized, the volumetric UHFAT outruns the NDAT (between V4 and V5). A similar activation pattern in LBBB canine hearts has been shown by Strik et al.^25^.

The relationship between NDAT and UHFAT shapes in the RBBB group is interestingly different. Volume activation, on average, precedes epicardial activation during the entire electrical depolarization. In RBBB, the LV, including the left part of the septum, is activated regularly through the left bundles, followed by the delayed activation of RV through the slow myocardial conduction towards the anterior, lateral wall, and finally to the outflow tract^16,26^. The physiological LV activation in RBBB is in agreement with regular LV activation in the Normal group depicted in leads V4–V6 (Fig. 2). The earlier UHFAT activation in V1 and V2 may be explained by the work of Sohi et al., who showed the delayed and shifted epicardial breakthrough on the right anterior chest in the RBBB depolarization pattern^27^.

**Figure 7:**
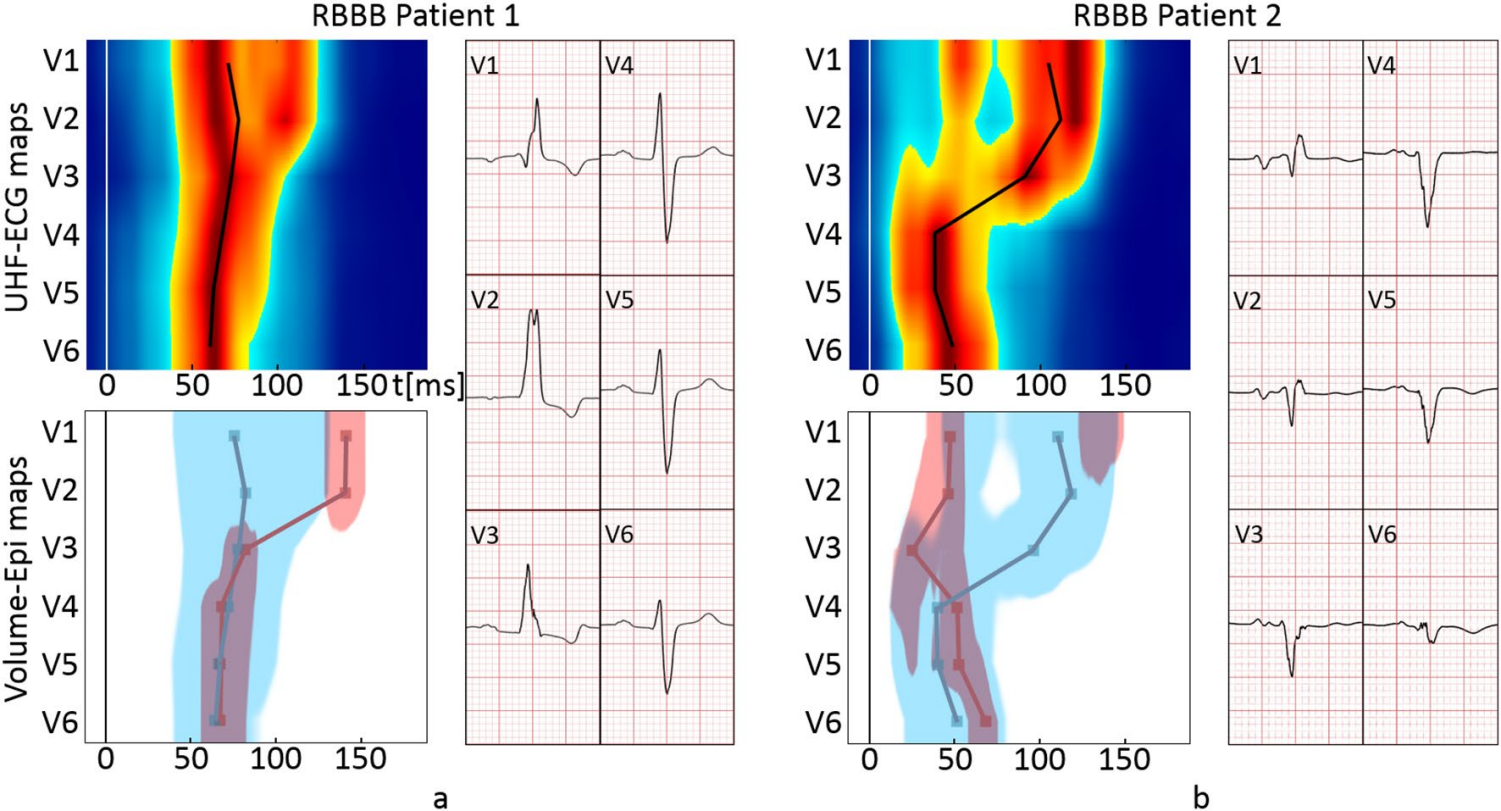
Examples of the instability of the RBBB depolarization shapes in V1–V3 created by the UHFAT and NDAT points. (**a**) UHFAT reflects the septal activation, while NDAT reflects the RV free wall activation in the V1–V3 leads. (**b**) Opposite example to (**a**). Abbreviations as in Table 2.

Despite the overall temporally dominating UHFAT activation in RBBB, an unstable behavior between UHFAT and NDAT occurs in some RBBB subjects within V1–V3 leads. These ECG leads cover the electrical action of the septum as well as the RV free wall. In the UHFAT map, which is spatial sensitive, when signals from the septum depolarization are stronger than signals from the right ventricular lateral wall, weaker signals from the later activated right ventricular lateral wall are suppressed by the earlier activated stronger septum^3^. In that case, the UHFAT point does not or does not fully reflect the later activated RV free wall even though the depolarization map indicates so (Fig. 7a). While the center of gravity assessing the UHFAT determines the volume activation well, it cannot reliably distinguish two activated volumes at different times under the V lead. Analogically, NDAT can express an early electrical activation in RBBB when the rSR’ shape in V1 is present, and the first negative slope is steeper than the second one (Fig. 7b). In these cases, a single numerical parameter partially fails, and the depolarization map should be visually inspected. To summarize, the lower agreement of the UHFAT and NDAT depolarization shapes in RBBB is the consequence of the near-field double electrical activation in V1–V3. This effect is well evident in the scatter plot of the RBBB group in Fig. 6.

#### Ventricular depolarization patterns during stimulation

In the studied groups with native ventricular conduction, the LV free wall is activated towards the epicardium (endo-to-epi) as the LV mass plays a strong role in favor of the volume activation (Fig. 2). LV-only stimulation by the LV lead, placed in CS from the epicardial side of the LV, can turn the depolarization wave in the “epi-to-endo” direction. The activation change is visible in the NIVCD patient (Fig. 3a,c) but is even more evident in the LBBB patient (Fig. 4a,c) as similarly shown in^25^. However, in the RBBB patient, the volumetric UHFAT still outruns the NDAT even during LV-only pacing under the LV lateral wall’s V leads (Fig. 5c). The explanation may lay in the LV lead placement. While the LV leads in NIVCD and LBBB patients are placed in the LV longitudinal mid-segment, the RBBB patient’s LV lead is placed in the basal segment, thus still far away from the V6 lead. The apicobasal position of the LV lead influences the positivity-negativity in the QRS complex in precordial leads^28^. Positive precordial QRS concordance suggests the basal location of the LV electrode, while negative V4–6 leads reveal a rather mid or apical LV lead position^29^. The position of the negative downslope in the QRS complex strongly affects the NDAT by the definition. The indication of the earliest NDAT activation followed by the UHFAT under the V6 with a markedly long delay from the pacing spike may suggest LV basal depolarization (Fig. 5c). The spatial distance between LV basal segment and most lateral V6 lead presumably plays a role here. Posterior precordial ECG electrodes (V7–8) could help better depict the electrical activity of the basal segments.

In all three CRT examples, RV-only pacing into the RV apex creates the overall LBBB-shaped character of the activation regardless of the native ventricular conduction pattern. The first activation occurs epicardially around the apex (V2–V4), followed by volumetric UHFAT activation and a very late activated LV lateral wall. We may speculate that the apical location of the pacing RV lead, together with the thinness of the RV wall against very closely placed V2–4 leads, favors fast epicardial NDAT activation with delayed endocardial wavefront as suggested by^23^.

### ND-ECG and UHF-ECG properties

The presented ND-ECG approach refers to intrinsic and intrinsicoid deflection studied in the past^9,12,30,31^ and recently similarly used by others^13,19,20^. ND-ECG depolarization analysis is technically possible from a single ECG beat, and the standard low-frequency clinical ECG can be sufficient for the analysis. UHF-ECG analysis requires a high dynamic and frequency range recording system and longer ECG measurement for QRS averaging (mostly at least 60 heartbeats). Nonetheless, in our previous work^5^, we demonstrated the feasibility of employing the UHF-ECG methodology for 10 s non-paced ECG strips with a sampling frequency of 1 kHz (QRS frequency content limited to up to 300 Hz). Next to the volumetric depolarization analysis, the UHF-ECG produces additional clinically relevant information about local activation duration^1,32,33^ under the V leads.

The temporal relationship between the QRS duration and UHF and ND depolarization times over the V leads is based on the fact that the QRS duration corresponds to the time of the earliest and latest amplitude deviation against an isoline from all the ECG leads. UHF and ND activation times are determined at the time point when a part of the myocardium is already depolarized or still waiting to be depolarized (Fig. 1). Since the UHF-ECG principle is based on the analysis of the UHF oscillations radiated from a ventricular segment and collected by the precordial electrode placed above this segment, the UHF-ECG does not discriminate the direction of the depolarizing breakthrough. ND-ECG relates to the closest epicardial activation based on the intrinsic deflection principle. However, the low-frequency nature of the ND-ECG method respects the different depolarization wavefronts and thus cannot exclude the influence of remote depolarization. Both described methods, but mainly their combination, could find their utilization generally in clinical cardiac pacing. For example, in pre-implant diagnostics, advanced information about the ventricular conduction pattern could help identify the most convenient pacing therapy or better select possible positive responders for CRT treatment. Because the UHF-ECG volume-epi depolarization maps can be created in real time, the information about the actual pacing effect can be provided almost immediately during the implant procedure. In post-implantation care is possible to perform subject-specific pacemaker settings optimization as well as clinical long-term follow-up evaluation of the ventricular depolarization pattern.

Further research is warranted, particularly in patients with myocardial fibrosis. It remains unclear to what extent of the scar or fibrotic tissue is sufficiently significant to impact the myocardial depolarization illustrated by the electrical depolarization map. Such investigations may contribute to comprehensive understanding of the clinical potential of the noninvasive volumetric-epicardial electrical activation approach.

## Conclusion

Our study first introduces the volumetric and epicardial ventricular electrical activation analysis by the combination of the two methodologically distant approaches using a standard 12-lead ECG electrode positions placement. We established the nd-DYS parameter describing the interventricular epicardial electrical dyssynchrony analogically to the previously presented volumetric e-DYS parameter. The epicardial nd-DYS significantly correlates with the volumetric e-DYS, where nd-DYS values are higher than e-DYS. Assessment of the spatio-temporal local differences between volumetric UHF-ECG and epicardial ND-ECG activation patterns permits a more detailed analysis of ventricular electrical activation, including the direction of the depolarization wave propagation in ventricles.

## Data Availability

We comply with the Scientific Reports data availability policy, and we will make data available upon reasonable request to the corresponding author (Pavel Leinveber).
